# Combination of twelve alleles at six quantitative trait loci determines grain weight in rice

**DOI:** 10.1371/journal.pone.0181588

**Published:** 2017-07-18

**Authors:** Yuxiang Zeng, Junsheng Shi, Zhijuan Ji, Zhihua Wen, Yan Liang, Changdeng Yang

**Affiliations:** 1 State Key Laboratory of Rice Biology, China National Rice Research Institute, Hangzhou, People’s Republic of China; 2 Seed Management Station of Zhengjiang Province, Hangzhou, People’s Republic of China; Institute of Genetics and Developmental Biology Chinese Academy of Sciences, CHINA

## Abstract

Grain weight, which is controlled by quantitative trait loci (QTLs), is one of the most important determinants of rice yield. Although many QTLs for grain weight have been identified, little is known about how different alleles in different QTLs coordinate to determine grain weight. In the present study, six grain-weight-QTLs were detected in seven mapping populations (two F_2_, one F_3_, and four recombinant inbred lines) developed by crossing ‘Lemont’, a United States *japonica* variety, with ‘Yangdao 4’, a Chinese *indica* variety. In each of the six loci, one allele from one parent increased grain weight and one allele from another parent decreased it. Thus, the 12 alleles at the six QTLs were subjected to regression analysis to examine whether they acted additively across loci leading to a linear relationship between the predicted breeding value of QTL and phenotype. Results suggested that a combination of the 12 alleles determined grain weight. In addition, plants carrying more grain-weight-increasing alleles had heavier grains than those carrying more grain-weight-decreasing alleles. This trend was consistent in the seven mapping populations. Thus, these six QTLs might be used in marker-assisted selection of grain weight, by stacking different grain-weight-increasing or -decreasing alleles.

## Introduction

Rice is a staple food crop for about half of the world’s population [1]. The improvement of rice yield is a permanent issue due to the rapid increase of global human population and to the decrease of arable land. Grain weight, one of the determinants of rice yield, is a typical quantitative trait controlled by quantitative trait loci (QTLs). Because grain weight is affected by grain length, width, and thickness, grain weight and grain shape are closely related traits. In the past two decades, a large number of QTLs responsible for rice grain weight or shape were identified [2] and some genes were cloned: *Ebisu Dwarf2* (*D2*) [3] and *D61* [4] on chromosome 1, *GW2* [5] on chromosome 2, *GS3* [6] and *qGL3* [7] on chromosome 3, *Dwarf11* (*D11*) [8], *GRAIN INCOMPLETE FILLING 1* (*GIF1*) [9], and *FLOURY ENDOSPERM2* (*FLO2*) [10] on chromosome 4, *Dwarf 1* (*D1*) [11], *GW5/qGW5* [12,13], *GS5* [14], and *SRS3* [15] on chromosome 5, *SMALL AND ROUND SEED1* (*SRS1/DEP2*) [16], *GW7/GL7* [17,18], and *GLW7* [19] on chromosome 7, *GW8* [20] on chromosome 8, and *Small And Round Seed 5* (*SRS5*) [21] and *Short Panicle1* (*SP1*) [22] on chromosome 11. The fine-mapped QTLs for grain shape include *GW1-1* [23], *GW1-2* [23], *qGRL1*.*1* [24], *qTGW1*.*1a* [25], and *qTGW1*.*1b* [25] on chromosome 1, *GS2* [26] on chromosome 2, *qGL-3a* [27], *gw3*.*1* [28], *GW3* [29], *TGW3b/SPP3b* [30], *qTGW3-1* [31], and *qTGW3*.*2* [32] on chromosome 3, *qGL4b* [33] and *Spr3* [34] on chromosome 4, *GW6* [29] and *spd6* [35] on chromosome 6, *qGL7* [36], *GS7* [37], and *qSS7* [38] on chromosome 7, *gw8*.*1* [39] on chromosome 8, *gw9*.*1* [40] on chromosome 9, and *tgw11* [41] on chromosome 11.

Occasionally, an allele at a specific QTL has a positive effect on grain shape while another has a negative effect. Theoretically, grain shape or weight can be manipulated by pyramiding different alleles with positive or negative effects on these traits. In rice breeding, many alleles have been mapped at different QTLs; however, which and how many alleles should be used in the marker-assisted selection of a desired phenotype are still important issues.

In a previous study, we developed two F_2_ and one F_3_ mapping populations by crossing ‘Lemont’, a United States sheath blight-susceptible *japonica* variety, with ‘Yangdao 4’, a Chinese sheath blight-resistant *indica* variety. The F_2_ and F_3_ mapping populations were initially used to identify the sheath blight-resistant QTL [42], not for mapping grain-weight-related QTLs. Six grain-weight-related QTLs were detected while analyzing the F_2_ and F_3_ populations, and we found that 12 alleles within these six grain-weight-QTLs acted cumulatively to control grain weight. In the present study, we used data from seven mapping populations, including the two F_2_ and the F_3_ populations developed in a previous study [42], and four recombinant inbred lines populations developed using the same parents, to elucidate how the 12 alleles in the six QTLs regulated grain weight and to test if these 12 alleles act additively across loci, leading to a linear relationship between the predictive breeding value of QTL and phenotype, or epistatically, leading to a nonlinear relationship between QTL values and phenotype.

## Materials and methods

### Mapping populations

‘Lemont’, a sheath blight-susceptible United States cultivar, was crossed with ‘Yangdao 4’, a relatively sheath blight-resistant Chinese cultivar, producing two F_2_, one F_3_, and four recombinant inbred line (RIL) populations (F_7_ to F_10_), which were used in the present study.

Detailed information on the two F_2_, the F_3_, and two of the RIL (F_7_ and F_8_) mapping populations is given in Zeng et al. [43]. Briefly, the first F_2_ mapping population (n = 190 individuals) was planted in May 2011 and the second F_2_ mapping population (n = 182 individuals) was sown in May 2012, both in Hangzhou, China; the F_3_ mapping population (n = 160 lines, each including 18 individuals that were arranged in three rows with six plants each), deriving from the former F_2_ populations, was planted in November 2012 in Hainan, China. The F_7_ and F_8_ RIL populations (n = 220 lines) were sown in May 2014 in Hangzhou and in November 2014 in Hainan, respectively.

The F_9_ RIL population (n = 220 lines) was planted in May 2015 at China National Rice Research Institute (CNRRI) farm in Hangzhou (119°95′E, 30°07′N) and the F_10_ RIL population (n = 220 lines) was sown in November 2015 at CNRRI trial station in Hainan (110°02′E, 18°48′N). Eighteen plants were grown from each of the F_7_, F_8_, F_9_ and F_10_ lines, arranged in three rows of six plants each, with 17 cm between plants and 20 cm between rows. Field management followed the common agronomic practices in Hangzhou or Hainan.

### Grain weight determination

Rice grains were sun-dried after harvest and stored at room temperature for at least one month before determining grain weight. One-hundred fully filled grains were randomly selected from the upper half of the panicles of each individual plant in the F_2_ populations and weighed using an electronic balance. As some *indica*/*japonica* hybrids were sterile, some of the individuals in the mapping populations did not have 100 fully filled grains; in this case, all available grains were used. In the F_3_, F_7_, and F_8_ populations, 50 fully filled grains were selected from 10 plants within each line (five grains per individual plant) and weighed. More than 100 fully filled grains from each line were measured in F_9_ and more than 80 fully filled grains from each line were measured in F_10_. The 50- to 100-grain weight data were converted to 1000-grain weight and used in the analysis.

### Marker assays and QTL analysis

The strategy for identifying grain-weight-related QTLs followed the method described in Zeng et al. [43]. Briefly, we first detected the QTLs responsible for grain weight using the three primary mapping populations (F_2_ and F_3_ populations), and then confirmed mapping results using four permanent mapping populations (F_7_ to F_10_).

One hundred and seventy nine polymorphic co-dominant markers covering 12 rice chromosomes were used to identify the QTLs responsible for grain weight in the F_2_ population planted in 2011 in Hangzhou and in the F_3_ population planted in 2012 in Hainan. Detailed information on these 179 markers is given in Wen et al. [42]. The six grain-weight-related QTLs detected in these two populations (see Results section) were further examined in the F_2_ populations planted in 2012 in Hangzhou, using 44 markers that covered the regions of these six QTLs, as described in Zeng et al. [43].

The six grain-weight-related QTLs yielded by the three primary mapping populations were further confirmed using the four RIL populations (F_7_ to F_10_) and 33 polymorphic markers (S1 Fig) that covered the regions of the six QTLs.

The multiple interval mapping (MIM) method was used to detect grain weight-related QTLs in the Windows QTL Cartographer 2.5 software (http://statgen.ncsu.edu/qtlcart/WQTLCart.htm), according to the models described in Zeng et al. [43]. The inclusive composite interval mapping (ICIM) [44, 45] was used to detect digenic epistatic loci using QTL IciMapping Version 3.2 software [46] based on 1000 permutations (*P* < 0.05). Digenic epistatic loci were also confirmed by two-way analysis of variance (ANOVA) using SAS 8.01 (SAS Institute, Cary, NC, USA).

### Statistical analyses

Shapiro-Wilk tests, two-way ANOVA, and linear regression analysis were performed in SAS 8.01 (SAS Institute, Cary, NC, USA).

## Results

### Frequency distribution of grain weight in seven mapping populations

**Fig 1:**
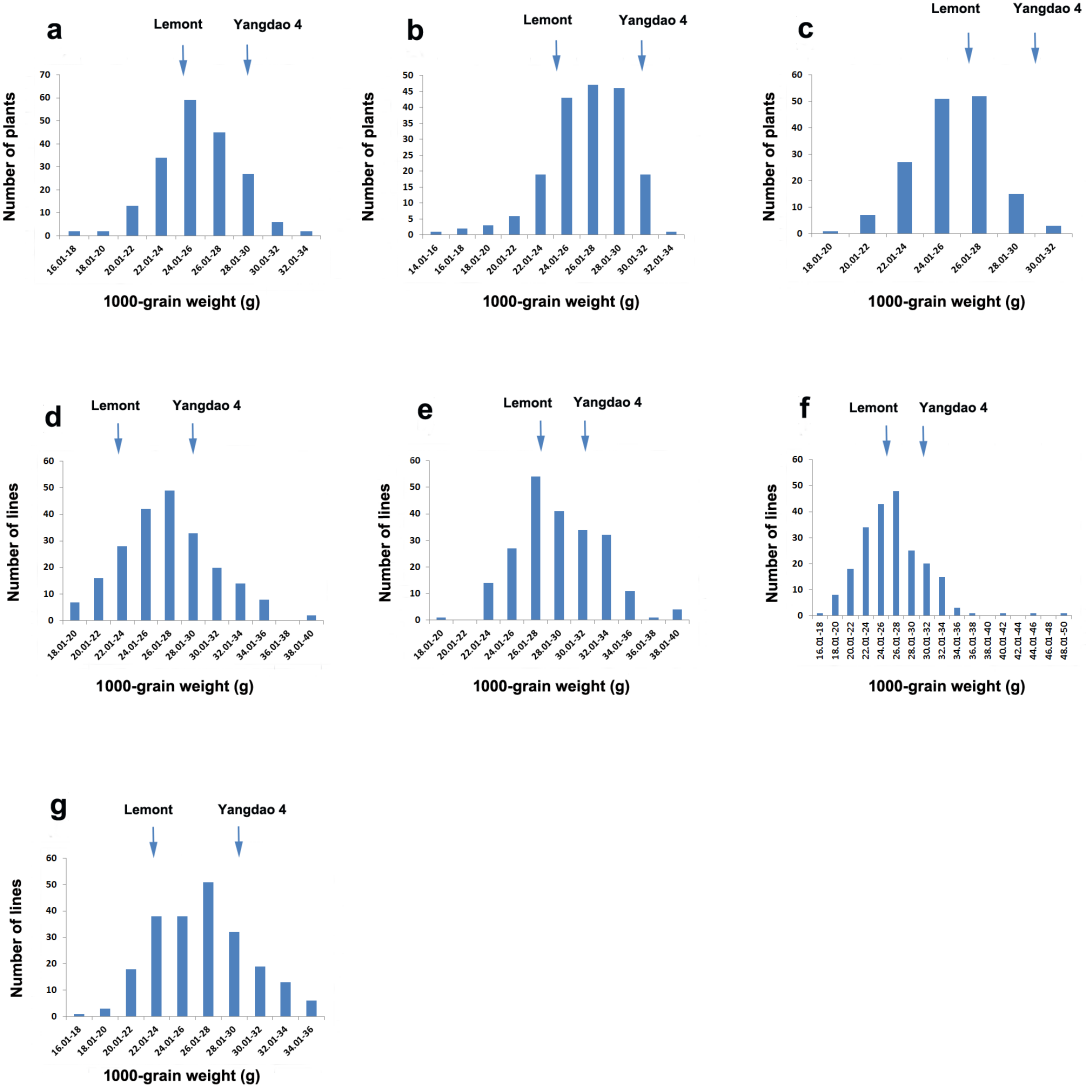
Frequency distribution of grain weight in the seven mapping populations derived from ‘Lemont’ × ‘Yangdao 4’ and grown under seven environments. (**a**) F_2_ population grown in 2011 in Hangzhou, (**b**) F_2_ population grown in 2012 in Hangzhou, (**c**) F_3_ population grown in 2012 in Hainan, (**d**) F_7_ recombinant inbred line (RIL) population grown in 2014 in Hangzhou, (**e**) F_8_ RIL population grown in 2014 in Hainan, (**f**) F_9_ RIL population grown in 2015 in Hangzhou, and (**g**) F_10_ RIL population grown in 2015 in Hainan.

The Shapiro-Wilk test run to check grain weight distribution in the seven mapping populations showed that data were normally distributed in three populations (F_2_ grown in 2011, W = 0.99, *P* = 0.14; F_2_ grown in 2012, W = 0.96, *P* < 0.01; F_3_, W = 0.99, *P* = 0.67; F_7_, W = 0.99, *P* = 0.03; F_8_, W = 0.98, *P* = 0.01; F_9_, W = 0.93, *P* < 0.01; and F_10_, W = 0.99, *P* = 0.32). Phenotypic data distribution in the seven mapping populations is shown in Fig 1.

### Grain-weight-QTLs in the primary mapping populations

The 179 polymorphic markers covering the 12 rice chromosomes used to detect the QTLs responsible for grain weight in the F_2_ population (n = 190 individuals) planted in 2011 in Hangzhou, revealed six QTLs (*qGW-1*, *qGW-3-1*, *qGW-3-2*, *qGW-4*, *qGW-7*, and *qGW-10*) on chromosomes 1, 3, 4, 7, and 10 (Table 1, S2 Fig). QTL analysis of the F_3_ population, deriving from F_2_ populations and planted in 2012 in Hainan (n = 160 lines), revealed that three of the six QTLs (*qGW-3-1*, *qGW-4*, and *qGW-10*) detected in F_2_ were present in F_3_ (Table 1, S3 Fig).

**Table 1:** Grain-weight-QTLs detected by multiple interval mapping in the seven mapping populations, derived from ‘Lemont’ × ‘Yangdao 4’ and planted in seven environments.

Mapping population (Mapping environment)	QTL name	Chr.	Marker interval^a^	Nearest marker	LR or LOD^b^	Additive effect^c^ (g)	Dominance effect	R^2^ (%)^d^
F_2_ (2011, Hangzhou)	*qGW-1*	1	D134B-D144A	D140A	20.20	1.11	-0.07	10.0
	*qGW-3-1*	3	D303-D309	D307	9.64	-0.78	0.42	3.6
	*qGW-3-2*	3	D336B-RM3585	D336B	30.28	1.39	-0.23	12.9
	*qGW-4*	4	D456-RM1113	D463	32.26	-1.35	-0.51	12.9
	*qGW-7*	7	D750-RM234	RM505	20.95	-1.02	0.15	8.2
	*qGW-10*	10	RM496-D1053	D1053	18.31	-0.96	-0.19	6.0
F_3_ (2012, Hainan)	*qGW-3-1*	3	RM232-D315	RM232	7.51	-2.31	1.44	10.1
	*qGW-4*	4	D456-RM1113	D463	12.85	-1.83	-0.24	8.1
	*qGW-10*	10	RM496-D1053	D1053	11.16	-1.66	-0.15	6.4
F_2_ (2012, Hangzhou)	*qGW-3-2*	3	D336B-RM3585	RM3585	10.87	1.42	-0.33	4.9
	*qGW-4*	4	D456-D463	D456	8.08	-1.49	1.38	5.8
	*qGW-10*	10	D1048-D1053	D1053	7.59	-1.07	1.09	3.5
F_7_ (2014, Hangzhou)	*qGW-1*	1	D140A-D144A	D140A	2.85	1.18	-	6.2
	*qGW-3-1*	3	D311-D315	D315	2.38	-1.24	-	5.2
	*qGW-3-2*	3	D335C-D336B	RM3684	6.76	1.56	-	12.0
	*qGW-4*	4	D456-D457B	D457B	5.36	-1.42	-	9.3
	*qGW-7*	7	D746-RM505	D746	4.77	-1.40	-	8.6
	*qGW-10*	10	D1042-RM496	D1048	3.23	-1.12	-	4.9
F_8_ (2014, Hainan)	*qGW-1*	1	D134B-D144A	D140A	3.08	1.07	-	5.2
	*qGW-3-1*	3	D309-D311	D311	2.61	-1.10	-	3.3
	*qGW-3-2*	3	D335C-D336B	RM3684	1.93	0.85	-	4.4
	*qGW-4*	4	D456-D457B	D456	6.43	-1.59	-	11.6
	*qGW-7*	7	D751-RM234	RM234	5.56	-1.43	-	9.0
	*qGW-10*	10	D1042-RM496	D1048	2.46	-0.98	-	4.0
F_9_ (2015, Hanghzou)	*qGW-1*	1	D134B-D144A	D140A	3.36	1.26	-	6.7
	*qGW-3-1*	3	D311-D315	D315	1.84	-1.16	-	4.0
	*qGW-3-2*	3	D335C-D336B	RM3684	5.11	1.46	-	10.3
	*qGW-4*	4	D456-D457B	D456	3.91	-1.34	-	7.7
	*qGW-7*	7	D746-RM505	D746	3.18	-1.33	-	6.7
	*qGW-10*	10	D1042-RM496	D1048	2.68	-1.22	-	5.2
F_10_ (2015, Hainian)	*qGW-1*	1	D140A-D144A	D140A	3.88	1.39	-	8.8
	*qGW-3-1*	3	D311-D315	D315	2.54	-1.15	-	5.0
	*qGW-3-2*	3	D335C-D336B	RM3684	4.24	1.18	-	7.1
	*qGW-4*	4	D456-D457B	D457B	5.93	-1.47	-	11.6
	*qGW-7*	7	D746-RM505	D746	4.57	-1.29	-	8.4
	*qGW-10*	10	D1042-RM496	D1048	2.12	-0.91	-	3.8

^a^Marker interval is defined by the flanking marker closest to the limit of the detection peak.

^b^LR values are presented for F_2_ and F_3_ populations, and LOD values are presented for F_7_, F_8_, F_9_ and F_10_ populations.

^c^Positive additive effects indicate that ‘Lemont’ increased grain weight and a negative additive effect indicated ‘Yangdao 4’ increased grain weight.

^d^Phenotypic variation explained by an individual QTL.

-, no data.

The six grain-weight-related QTLs resolved for F_2_ and F_3_ populations (Table 1) were further examined in the F_2_ mapping population (n = 182 individuals) planted in 2012 in Hangzhou. The 44 markers covering the regions of these six QTLs used to analyze this F_2_ population detected three QTLs: *qGW-3-2*, *qGW-4*, and *qGW-10* on chromosomes 3, 4, and 10 (Table 1, S4 Fig).

### Grain-weight-QTLs confirmation using RIL populations

The six grain-weight-QTLs detected in the three primary mapping populations were further confirmed in the four RIL populations (n = 220 lines) using 33 polymorphic markers that covered the regions of the six QTLs. These 33 markers represented 274.8 cM, with an average of 10.2 cM between adjacent markers (S1 Fig). All the six grain-weight-QTLs identified in the three primary mapping populations were detected in the four RIL populations (Table 1, S5 to S8 Figs).

### QTLs-by-environment and QTLs-by-population interactions

Using a two-way ANOVA, we examined if the six grain-weight-QTLs significantly interacted with environments or mapping populations. The closest markers to the six QTLs in each mapping population were used to represent the QTL genotypes. Only three significant QTLs were detected in the F_3_ population planted in 2012 in Hainan and in the F_2_ population planted in 2012 in Hangzhou (Table 1). We used multiple interval mapping to identify the closest markers to the other three putative grain-weight-QTLs in these two populations, and the heterozygotes of F_2_ populations were omitted from the following two-way ANOVA.

No significant interactions were detected between each of the six grain-weight-QTLs and environment (Table 2), suggesting that the effects of these QTLs were stable across the different mapping environments. In addition, no significant interactions were detected between grain-weight-QTLs and populations, except for *qGW-7* (Table 3). This is in agreement with the fact that *qGW-7* was not detected in two populations: it was present in five populations (Table 1).

**Table 2:** Quantitative trait loci (QTLs)-by-environment interactions examined using two-way analysis of variance. As seven mapping environments were considered (three primary and four recombinant inbred line mapping populations), there were six degrees of freedom. Because there were two genotypes at each QTL (heterozygotes were omitted and not used in the analysis), the degree of freedom for QTL was 1.

QTL by environment interaction	Degree of freedom	Type I sum of squares	Mean square	F value	*P*
Environment	6	1433.28	238.88	19.02	<0.01
QTL	1	978.16	978.16	77.90	<0.01
*qGW-1* × Environment	6	37.29	6.21	0.49	0.81
Environment	6	1292.87	215.48	15.94	<0.01
QTL	1	102.99	102.99	7.62	<0.01
*qGW-3-1* × Environment	6	44.80	7.47	0.55	0.77
Environment	6	1461.80	243.63	19.93	<0.01
QTL	1	1437.97	1437.97	117.63	<0.01
*qGW-3-2*× Environment	6	126.45	21.07	1.72	0.11
Environment	6	1266.74	211.12	17.50	<0.01
QTL	1	1404.27	1404.27	116.38	<0.01
*qGW-4* × Environment	6	3.96	0.66	0.05	0.99
Environment	6	1368.39	228.07	18.17	<0.01
QTL	1	652.12	652.12	51.96	<0.01
*qGW-7*× Environment	6	114.64	19.11	1.52	0.17
Environment	6	1309.43	218.24	16.59	<0.01
QTL	1	398.59	398.59	30.30	<0.01
*qGW-10* × Environment	6	71.87	11.98	0.91	0.49

**Table 3:** Quantitative trait loci (QTLs)-by-population interactions examined using two-way analysis of variance. As there were three types of mapping populations (F_2_, F_3_, and recombinant inbred line populations), there were two degrees of freedom. There were two genotypes at each QTL (heterozygotes were omitted and not used in analysis) and the degree of freedom for QTL was 1.

QTL by population interaction	Degree of freedom	Type I sum of squares	Mean square	F value	*P*
Population	2	416.00	208.00	15.54	<0.01
QTL	1	989.36	989.36	73.94	<0.01
*qGW-1* × Population	2	21.11	10.56	0.79	0.45
Population	2	262.89	131.44	9.09	<0.01
QTL	1	65.11	65.11	4.50	0.03
*qGW-3-1* × Population	2	0.92	0.46	0.03	0.97
Population	2	424.37	212.19	16.16	<0.01
QTL	1	1448.09	1448.09	110.26	<0.01
*qGW-3-2* × Population	2	44.89	22.44	1.71	0.18
Population	2	273.73	136.87	10.56	<0.01
QTL	1	1290.74	1290.74	99.56	<0.01
*qGW-4* × Population	2	0.53	0.27	0.02	0.98
Population	2	403.22	201.61	15.21	<0.01
QTL	1	711.27	711.27	53.67	<0.01
*qGW-7* × Population	2	123.81	61.91	4.67	<0.01**
Population	2	356.19	178.09	12.78	<0.01
QTL	1	400.67	400.67	28.76	<0.01
*qGW-10* × Population	2	62.89	31.45	2.26	0.11

**Values were highly significant at *P* < 0.01.

### Digenic epistasis in the seven mapping populations

Digenic epistatic loci were detected using inclusive composite interval mapping (ICIM) and the QTL IciMapping software, and their significance was further confirmed by two-way ANOVA. No significant digenic epistatic QTLs were detected in the F_2_ population planted in 2011 in Hangzhou, using the 179 markers that covered the 12 rice chromosomes. Ten pairs of digenic epistatic loci were found in the F_3_ population that derived from this F_2_ population (Table 4) and seven of them were significant, according to the two-way ANOVA performed using one of the flanking markers to represent the loci (S1 Table). One pair of digenic epistatic loci was detected in the F_2_ population grown in 2012 in Hangzhou (Table 5) using 44 markers, and this was confirmed significant by the two-way ANOVA (S2 Table). No significant digenic epistatic loci were detected in the RIL populations, probably because only 33 markers were used to genotype these populations. These results suggested that epistasis is involved in the regulation of grain weight (Tables 4 and 5), although no significant interactions were detected among the six grain-weight-related QTLs (*qGW-1*, *qGW-3-1*, *qGW-3-2*, *qGW-4*, *qGW-7*, and *qGW-10*).

**Table 4:** Pairs of digenic epistatic loci detected in the F_3_ population, derived from ‘Lemont’ × ‘Yangdao 4’, and grown in 2012 in Hainan, by using inclusive composite interval mapping.

Chr. 1	Left Marker 1	Right Marker 1	Chr. 2	Left Marker 2	Right Marker 2	LOD	PVE (%)	Add1	Add2	Dom1	Dom2	Add by Add	Add by Dom	Dom by Add	Dom by Dom
1	D108C	RM1201	7	RM3404	RM182	5.37	12.63	0.58	-0.06	0.12	0.55	-0.51	-2.10	-0.17	0.06
1	D122E	D128A	4	D436	D440	5.95	10.09	-1.10	0.62	0.85	0.32	0.73	1.06	-1.47	-1.23
1	D128A	RM1297	6	RM1340	D643	5.12	9.74	-0.90	0.92	1.30	1.41	-0.42	0.88	-1.24	-1.62
2	D205	D208	9	D927	RM409	5.50	11.79	-0.62	-0.13	-0.54	-0.69	-1.31	0.63	0.23	1.26
2	RM3685	D236	3	D336B	RM3585	5.01	12.14	1.51	2.21	-0.20	0.31	-0.35	-0.87	-1.90	0.51
3	D301	D303	9	D933	RM7424	5.29	10.09	-0.50	-0.52	0.06	-0.40	-0.25	1.08	1.44	-0.53
3	D307	D309	4	D440	D444	5.15	9.95	-0.57	0.45	1.17	0.50	-0.56	-0.48	-1.40	-1.44
3	D331B	RM5813	11	D1133	D1142	5.54	9.77	-1.56	-0.67	0.87	0.83	-1.17	1.80	0.60	-1.47
6	RM1340	D643	11	D1133	D1142	5.33	12.35	0.95	-0.55	0.59	0.17	1.74	-0.72	0.70	-0.14
11	RM26155	D1113	11	D1133	D1142	6.73	13.62	1.20	-1.08	2.03	2.19	0.96	-1.87	1.24	-3.72

Chr. 1 and Chr. 2 are the first and second chromosome positions of the detected digenic epistatic loci, respectively. LOD: logarithm of odds. PVE (%): phenotypic variation explained by digenic epistatic interaction. Add1: estimated additive effect of the first digenic locus. Add2: estimated additive effect of the second digenic locus. Dom1: estimated dominance effect of the first digenic locus. Dom2: estimated dominance effect of the second digenic locus. Add by Add: additive-by-additive digenic interaction. Add by Dom: additive-by-dominance digenic interaction. Dom by Add: dominance-by-additive digenic interaction. Dom by Dom: dominance-by-dominance digenic interaction.

**Table 5:** The pair of digenic epistatic loci detected in the F_2_ population, derived from ‘Lemont’ × ‘Yangdao 4’ and grown in 2012 in Hangzhou, using inclusive composite interval mapping.

Chr.1	Left Marker 1	Right Marker 1	Chr.2	Left Marker 2	Right Marker 2	LOD	PVE (%)	Add1	Add2	Dom1	Dom2	Add by Add	Add by Dom	Dom by Add	Dom by Dom
12	D1252	RM1246	7	D755	D760	5.43	12.48	-0.65	-1.20	-1.24	-0.89	0.53	1.18	1.61	2.64

Chr. 1 and Chr. 2 are the first and second chromosome positions of the detected digenic epistatic loci, respectively. LOD: logarithm of odds. PVE (%): phenotypic variation explained by digenic epistatic interaction. Add1: estimated additive effect of the first digenic locus. Add2: estimated additive effect of the second digenic locus. Dom1: estimated dominance effect of the first digenic locus. Dom2: estimated dominance effect of the second digenic locus. Add by Add: additive-by-additive digenic interaction. Add by Dom: additive-by-dominance digenic interaction. Dom by Add: dominance-by-additive digenic interaction. Dom by Dom: dominance-by-dominance digenic interaction.

### Regulation of grain weight by 12 alleles in six QTLs

Based on the QTLs-by-environment interaction analysis, the effects of the six grain-weight-QTLs were stable across the different environments. These six QTLs did not significantly interact, according to ICIM results. We further analyzed the coordination between the different alleles from these six QTLs regulating grain weight.

In each of the six QTLs (*qGW-1*, *qGW-3-1*, *qGW-3-2*, *qGW-4*, *qGW-7*, and *qGW-10*), there was an allele from one parent that increased grain weight and one allele from the other parent that decreased it. The six grain-weight-increasing alleles were *qGW-1LE*, *qGW-3-1YD*, *qGW-3-2LE*, *qGW-4YD*, *qGW-7YD*, and *qGW-10YD*, and the six grain-weight-decreasing alleles were *qGW-1YD*, *qGW-3-1LE*, *qGW-3-2YD*, *qGW-4LE*, *qGW-7LE*, and *qGW-10LE*; ‘*LE*’ or ‘*YD*’ suffixes in QTL names indicate the allele was inherited from the ‘Lemont’ or the ‘Yangdao 4’ parent, respectively.

A linear regression analysis was performed to examine if the 12 alleles in the six QTLs acted additively across loci leading to a linear relationship between the predicted breeding values of QTLs and the phenotype. Such a linear relationship implies that plants carrying more grain-weight-increasing alleles have heavier grains than those carrying more grain-weight-decreasing alleles. We first calculated the genotypic value of each individual plant (or line) in the seven mapping populations, which was used as the predictive breeding value of each individual plant or line. The genotypic value of an individual plant or line was calculated by adding the estimated additive effects of each of the six QTLs in the RIL populations. A positive additive effect was used if a locus carried a grain-weight-increasing allele, and a negative additive effect was used if a locus carried a grain-weight-decreasing allele. For the F_2_ mapping populations, the genotypic value of an individual plant was calculated by adding the estimated additive effects and dominance effects of each of the six QTLs. The dominance effects of heterozygotes were summed to the additive effects of the homozygotes across the six loci in the F_2_ populations. The additive or dominance effects of the six QTLs are listed in Table 1. Because only three significant QTLs were detected in the F_3_ population planted in 2012 in Hainan and in the F_2_ population planted in 2012 in Hangzhou, the additive or dominance effects of the other three putative QTLs were resolved by the MIM method using Windows QTL Cartographer 2.5.

**Fig 2:**
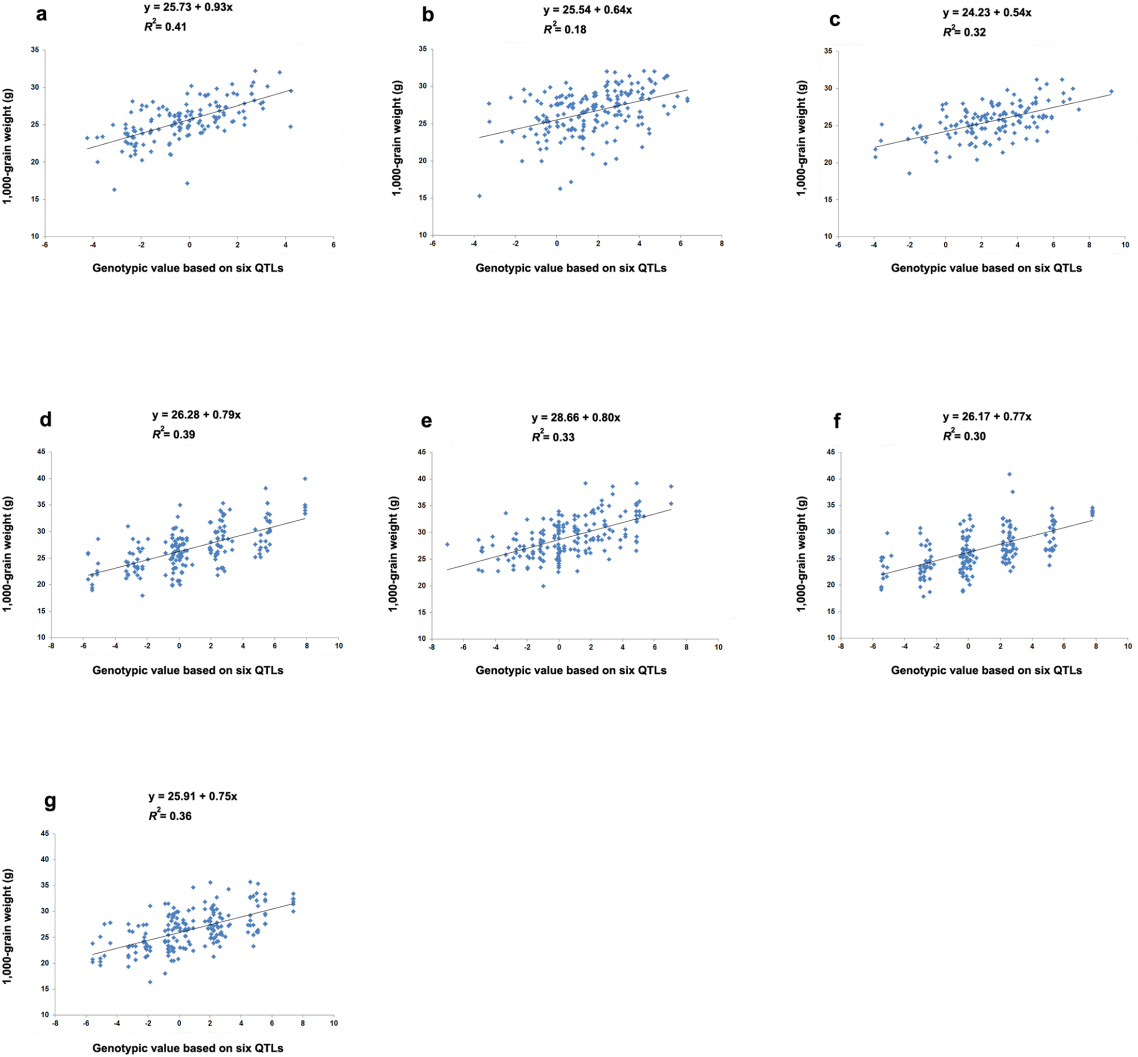
Linear regression analysis between 1000-grain-weight and genotypic values of individual plants/lines in the seven mapping populations (two F_2_, one F_3_, and four recombinant inbred line (RIL) populations), derived from ‘Lemont’ × ‘Yangdao 4’ and grown under different environmental conditions. (**a**) F_2_ population grown in 2011 in Hangzhou, (**b**) F_2_ population grown in 2012 in Hangzhou, (**c**) F_3_ population grown in 2012 in Hainan, (**d**) F_7_ RIL population grown in 2014 in Hangzhou, (**e**) F_8_ RIL population grown in 2014 in Hainan, (**f**) F_9_ RIL population grown in 2015 in Hangzhou, and (**g**) F_10_ RIL population grown in 2015 in Hainan. The genotypic value of each individual plant/line was calculated by adding the estimated additive effects of the six quantitative trait loci (QTLs) in the RIL populations, or by adding the estimated additive effects and dominance effects of the six QTLs in the F_2_ populations. The dominance effects of the heterozygotes were summed to the additive effects of the homozygotes across the six loci to determine QTL genotypic values in the F_2_ populations. When calculating genotypic values, a positive additive effect was used if a locus carried a grain-weight-increasing allele, and a negative additive effect was used if a locus carried a grain-weight-decreasing allele.

Linear regression analysis between the genotypic value and grain weight of all individual plants or lines in the seven mapping populations (Fig 2) revealed significant relationships (F_2_ grown in 2011, F = 96.95, *P* < 0.0001; F_2_ grown in 2012, F = 41.36, *P* < 0.0001; F_3_, F = 65.42, *P* < 0.0001; F_7_, F = 120.14, *P* < 0.0001; F_8_, F = 92.02, *P* < 0.0001; F_9_, F = 79.39, *P* < 0.0001, and F_10_, F = 103.74, *P* < 0.0001). The coefficient of determination (R^2^) was used as an estimate of the cumulative heritability of the six QTLs, revealing it was 41%, 18%, 32%, 39%, 33%, 30%, and 36% for the F_2_ grown in 2011, F_2_ grown in 2012, F_3_, F_7_, F_8_, F_9_, and F_10_, respectively. These results demonstrated that the 12 alleles in the six QTLs acted additively in grain weight regulation. Thus, plants carrying more grain-weight-increasing alleles had heavier grains than those carrying more grain-weight-decreasing alleles.

## Discussion

Seven mapping populations (two F_2_, one F_3_, and four RIL) and three genetic linkage maps were used in the present study to identify the QTLs responsible for rice grain weight. A common linkage map generated with 179 markers, which covered the 12 rice chromosomes, was used to detect QTLs in the F_2_ population planted in 2011 in Hangzhou and in its descendant F_3_ population planted in 2012 in Hainan. This yielded six grain-weight-QTLs, which were further examined in a second F_2_ population planted in 2012 in Hangzhou. To save time and labor, we did not analyze this population with the 179 markers covering the 12 chromosomes. Instead, we constructed a linkage map with 44 markers covering the regions of the grain-weight-QTLs detected in the F_2_ and F_3_ populations.

The six QTLs detected in the three primary mapping populations were further confirmed using four RIL populations, using a common linkage map constructed with 33 markers that covered the locations of these QTLs. This method saved time and labor but some of the grain-weight-QTLs with minor effect might have been neglected, as only two of the seven mapping populations were analyzed with markers covering all 12 chromosomes. Still, this strategy was successfully used for identifying the QTLs responsible for grain length in a previous study [43] and, because we could confirm the QTLs detected in the present study, we believe this is a reliable method for studying relevant traits concerning rice grain shape or weight.

**Fig 3:**
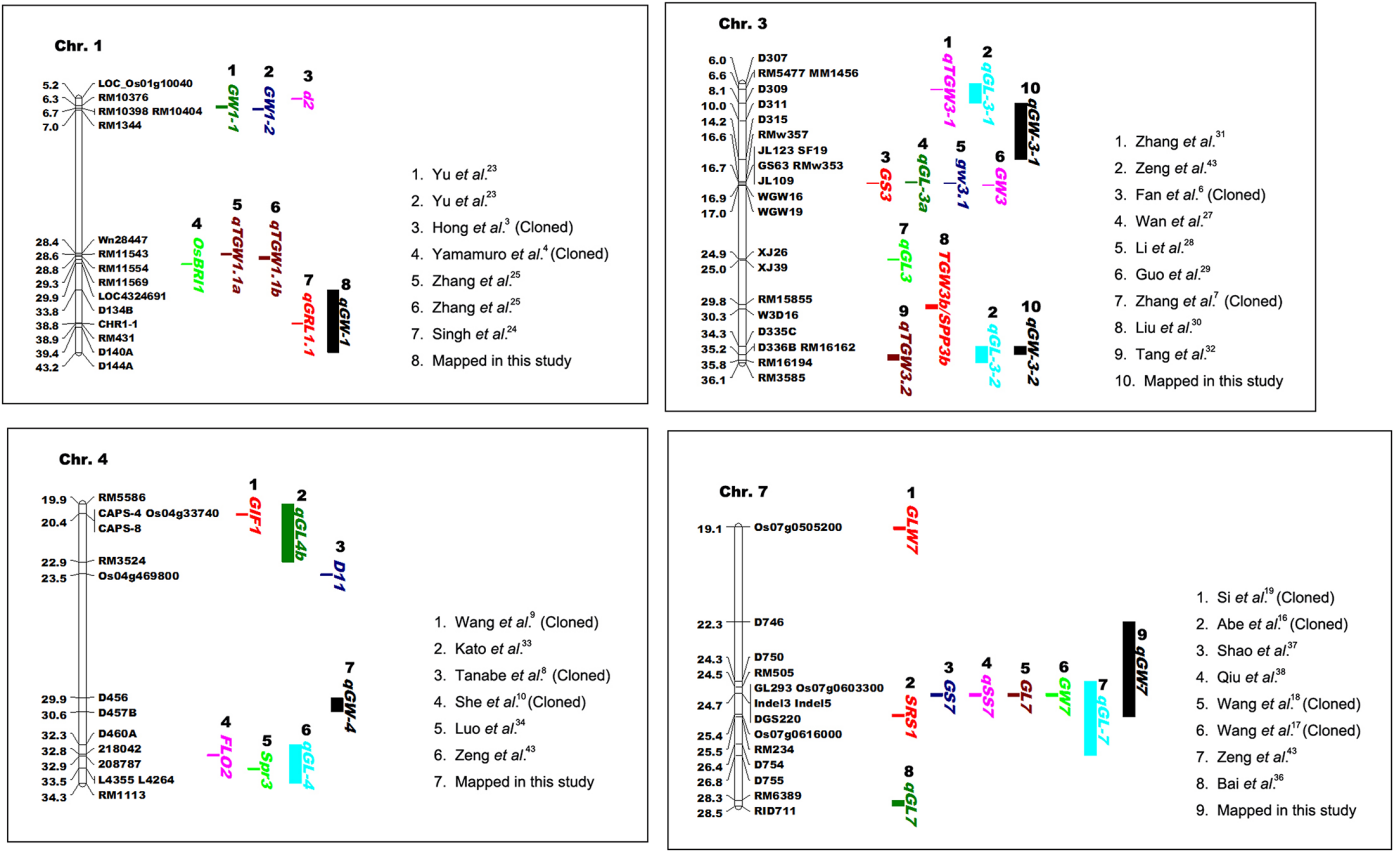
Comparison of the grain-shape- or grain-weight-related genes mapped on chromosomes 1, 3, 4, and 7 in the present and previous studies. Numbers to the left of the chromosome bar indicate the physical position (Mb) of the corresponding markers. The physical positions of the markers were determined using the basic local alignment search tool (BLAST) on the National Center for Biotechnology Information (NCBI) website (https://www.ncbi.nlm.nih.gov/) and the Nipponbare (IRGSP-1.0) as the reference sequences.

To compare the positions of the QTLs found in the present and in previous studies, we first determined the largest marker-flanking intervals of the six grain-weight-QTLs according to the mapping results in the four RIL populations (Table 1), as we believed that the results were more accurate in RIL than in F_2_ or F_3_. The largest marker intervals for *qGW-1*, *qGW-3-1*, *qGW-3-2*, *qGW-4*, *qGW-7*, and *qGW-10* were D134B-D144A, D309-D315, D335C-D336B, D456-D457B, D746-RM234, and D1042-RM496, respectively. The genes/QTLs responsible for grain shape or grain weight that were cloned or fine mapped on chromosomes 1, 3, 4, and 7 in previous studies were illustrated and compared with the QTLs detected in the present study (Fig 3). Chromosome 10 was not illustrated because no grain-shape-QTLs or grain-weight-QTLs were cloned or fine mapped on this chromosome. There were no QTLs co-localized with *qGW-4* or *qGW-10* but *qGW-1* was co-localized with *qGRL1*.*1*, a QTL identified by Singh et al. [24]. However, it is not clear whether the two QTLs are in the same gene. The QTL *qGW-3-1* was adjacent to *qGL-3-1*, a grain-length-QTL that we previously identified using the same ‘Lemont’ × ‘Yangdao 4’ mapping populations [43]. It is possible that *qGW-3-1* and *qGL-3-1* are in the same gene, but further studies are needed to confirm this. The *qGW-3-2* was co-localized with *qTGW3*.*2* [32] and *qGL-3-2* [43], the latter being a grain-length-related QTL identified by us in a previous study using the same mapping populations. Thus, *qGW-3-2* and *qGL-3-2* might be in the same gene. The *qGW-7* was co-localized with *SRS1* [16], *GL7/GW7* [17,18], *GS7* [37], *qSS7* [38], and *qGL-7* [43]. Although *SRS1* [16] and *GL7/GW7* [17,18] were cloned, it is not clear if *qGW-7* is allelic to either or both of them, based on the available information.

Grain weight is one of the most important determinants of rice yield. Because it directly influences grain yield, it has attracted great attention within the rice genetic research community. A large number of QTLs responsible for grain weight have been reported in previous studies, but it is still not clear how the different alleles in the several grain-weight-QTLs coordinated to determine grain weight. In the present study, we found an allele increasing and another decreasing grain weight within each of the six grain-weight-QTLs identified. We also found that plants carrying more grain-weight-increasing alleles had heavier grains than those carrying more grain-weigh-decreasing alleles. Regression analysis indicated that the 12 alleles in the six QTLs acted additively across loci, leading to a linear relationship between genotypic values and phenotype. Given that these six QTLs did not interact with each other, they might be involved in six independent pathways. Although the six QTLs detected in this study were mapped in relatively rough marker intervals, the closest markers to these QTLs can be used in marker-assisted breeding to pyramid the number of alleles needed to form a desired phenotype. However, caution must be taken because it is not known if the effect of the 12 alleles from the six QTLs is maintained when genes are introduced into different genetic backgrounds.

## Supporting information

S1 Fig The 33 polymorphic markers used in the QTL analysis of the four RIL populations. Numbers at the left of the chromosome bars indicate the genetic positions (cM) of the corresponding markers.
(TIF)

S2 Fig LOD score curves of the QTLs for grain weight detected in the F_2_ population grown in 2011 in Hangzhou. (TIF)

S3 Fig LOD score curves of the QTLs for grain weight detected in the F_3_ population grown in 2012 in Hainan. (TIF)

S4 Fig LOD score curves of the QTLs for grain weight detected in the F_2_ population grown in 2012 in Hangzhou. (TIF)

S5 Fig LOD score curves of the QTLs for grain weight detected in the F_7_ RIL population grown in 2014 in Hangzhou. (TIF)

S6 Fig LOD score curves of the QTLs for grain weight detected in the F_8_ RIL population grown in 2014 in Hainan. (TIF)

S7 Fig LOD score curves of the QTLs for grain weight detected in the F_9_ RIL population grown in 2015 in Hangzhou. (TIF)

S8 Fig LOD score curves of the QTLs for grain weight detected in the F_10_ RIL population grown in 2015 in Hainan. (TIF)

S1 Table Two-way analysis of variance used to confirm the digenic epistatic loci detected in the F_3_ population, derived from ‘Lemont’ × ‘Yangdao 4’, and grown in 2012 in Hainan, using inclusive composite interval mapping. DF: degrees of freedom; SS: sum of squares; *, *P* < 0.05; **, *P* < 0.01.
(DOCX)

S2 Table Two-way analysis of variance used to confirm the digenic epistatic loci detected in the F_2_ population, derived from ‘Lemont’ × ‘Yangdao 4’, and grown in 2012 in Hangzhou, using inclusive composite interval mapping. DF: degrees of freedom; SS: sum of squares; *, *P* < 0.05; **, *P* < 0.01.
(DOCX)
