# Supplementary figures and images for: Combination of twelve alleles at six quantitative trait loci determines grain weight in rice
Source: PLoS One. 2017 Jul 18;12(7):e0181588. doi: 10.1371/journal.pone.0181588 (PMC5515452; doi:10.1371/journal.pone.0181588)

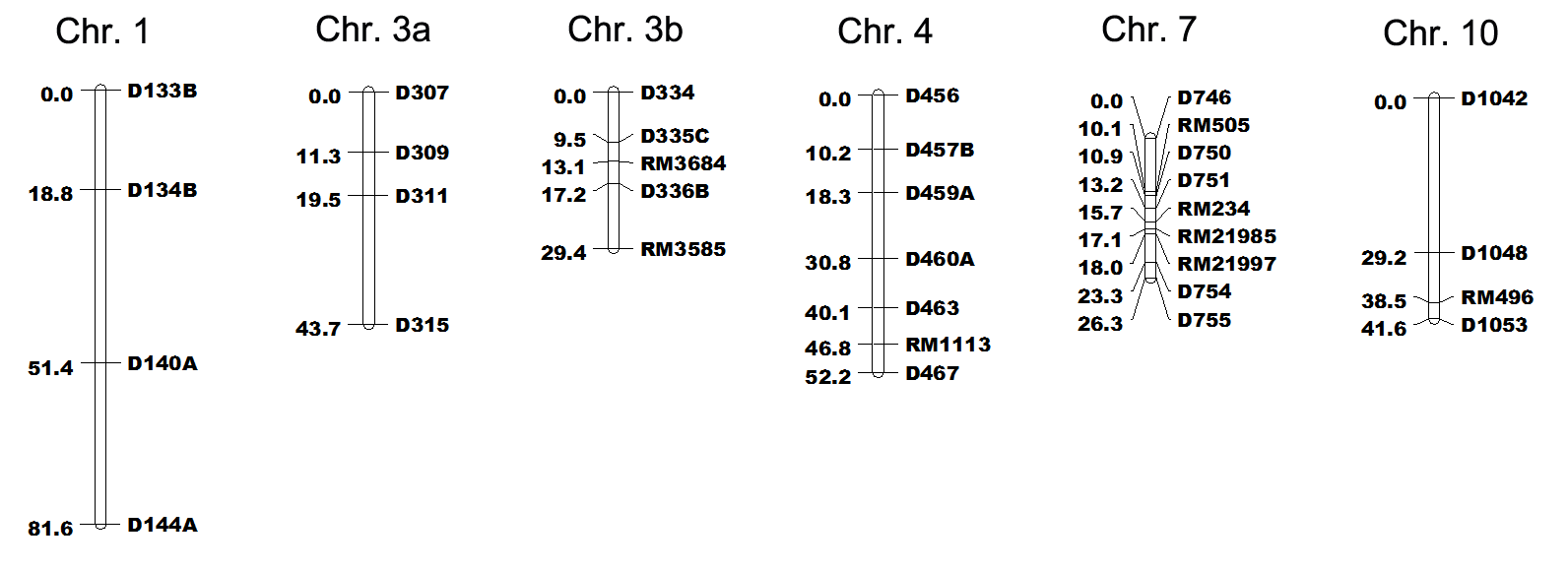

Supplement: S1 Fig — Numbers at the left of the chromosome bars indicate the genetic positions (cM) of the corresponding markers. (TIF) [file pone.0181588.s001.tif]

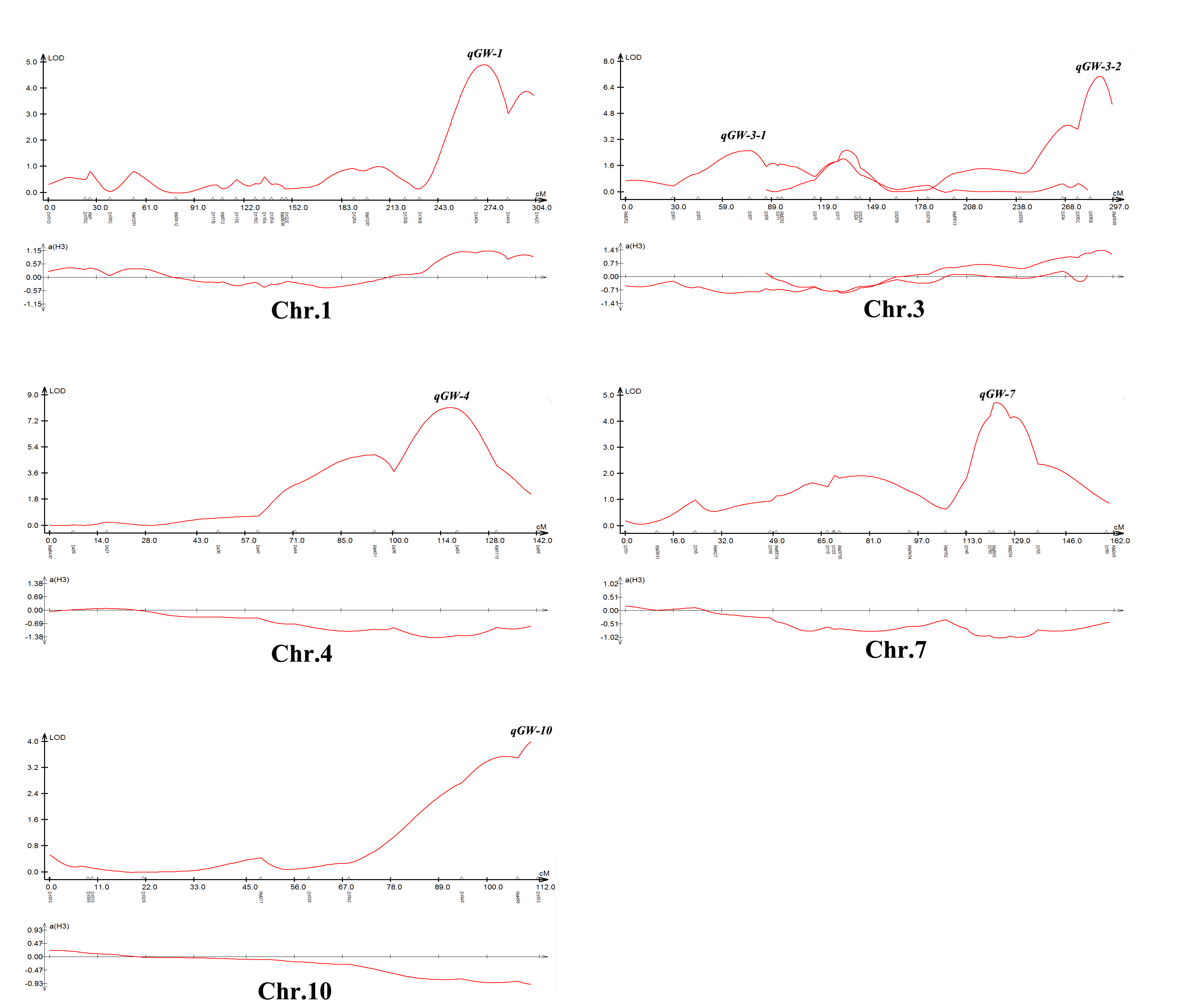

Supplement: S2 Fig — (TIF) [file pone.0181588.s002.tif]

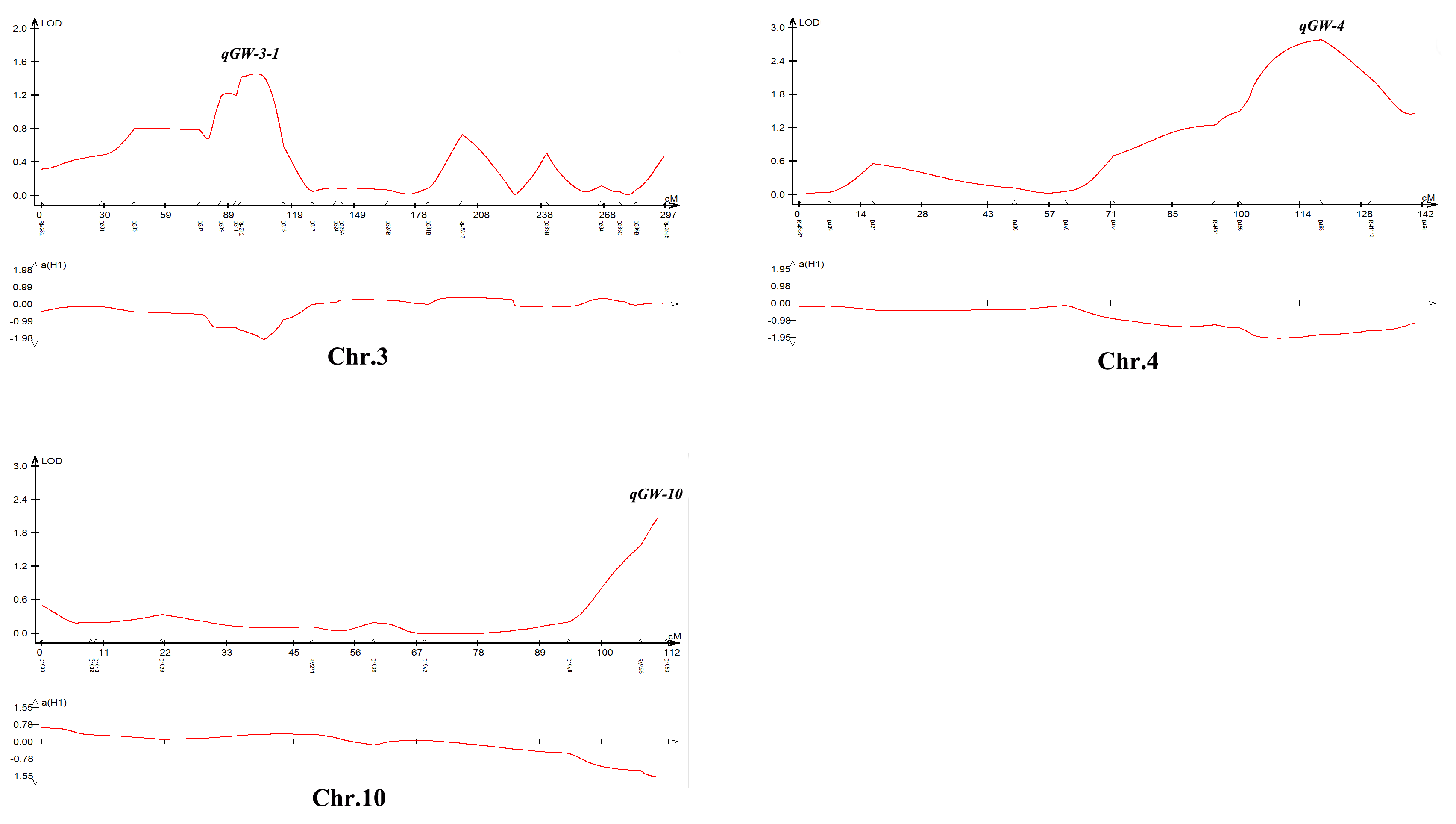

Supplement: S3 Fig — (TIF) [file pone.0181588.s003.tif]

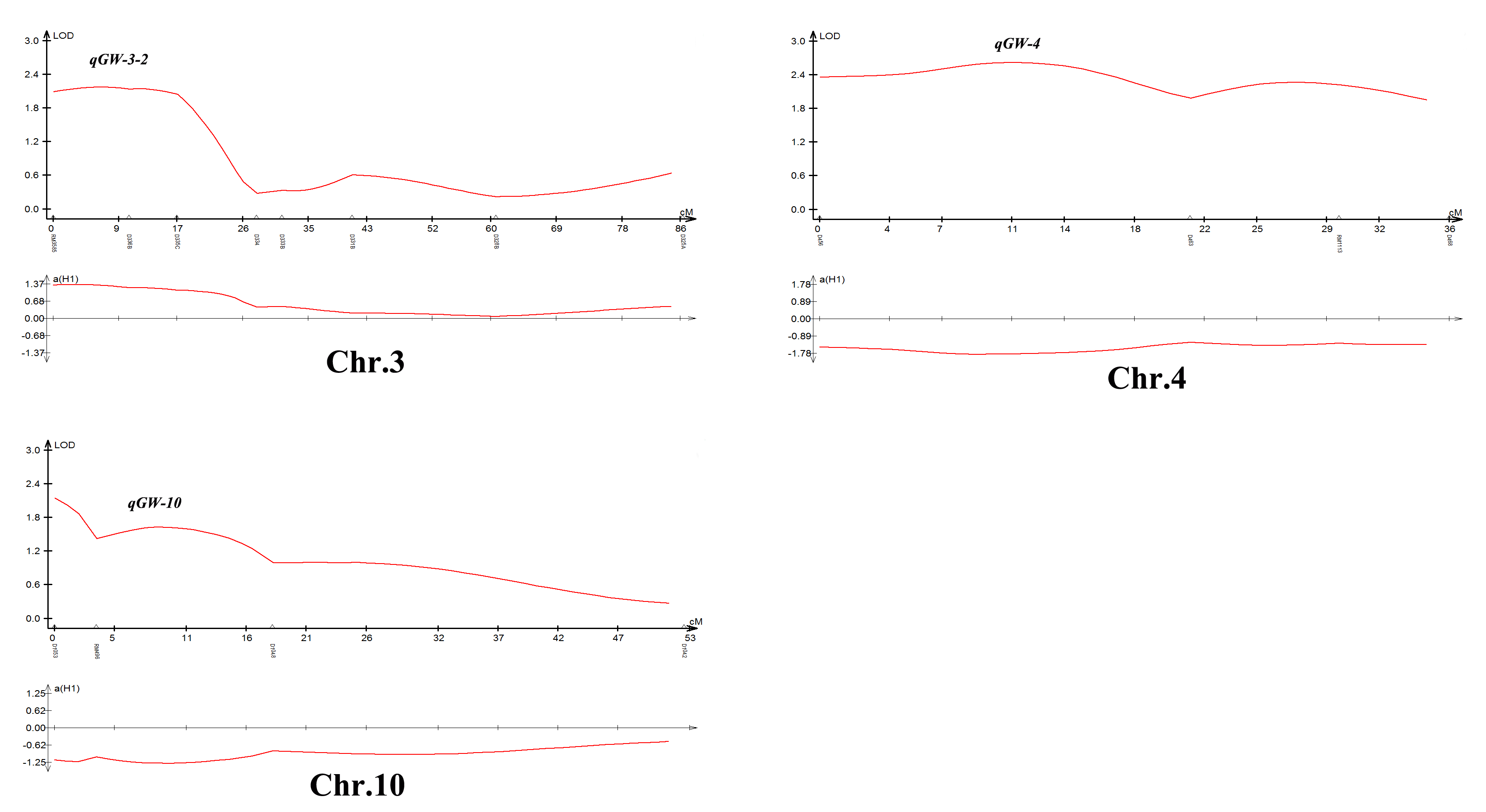

Supplement: S4 Fig — (TIF) [file pone.0181588.s004.tif]

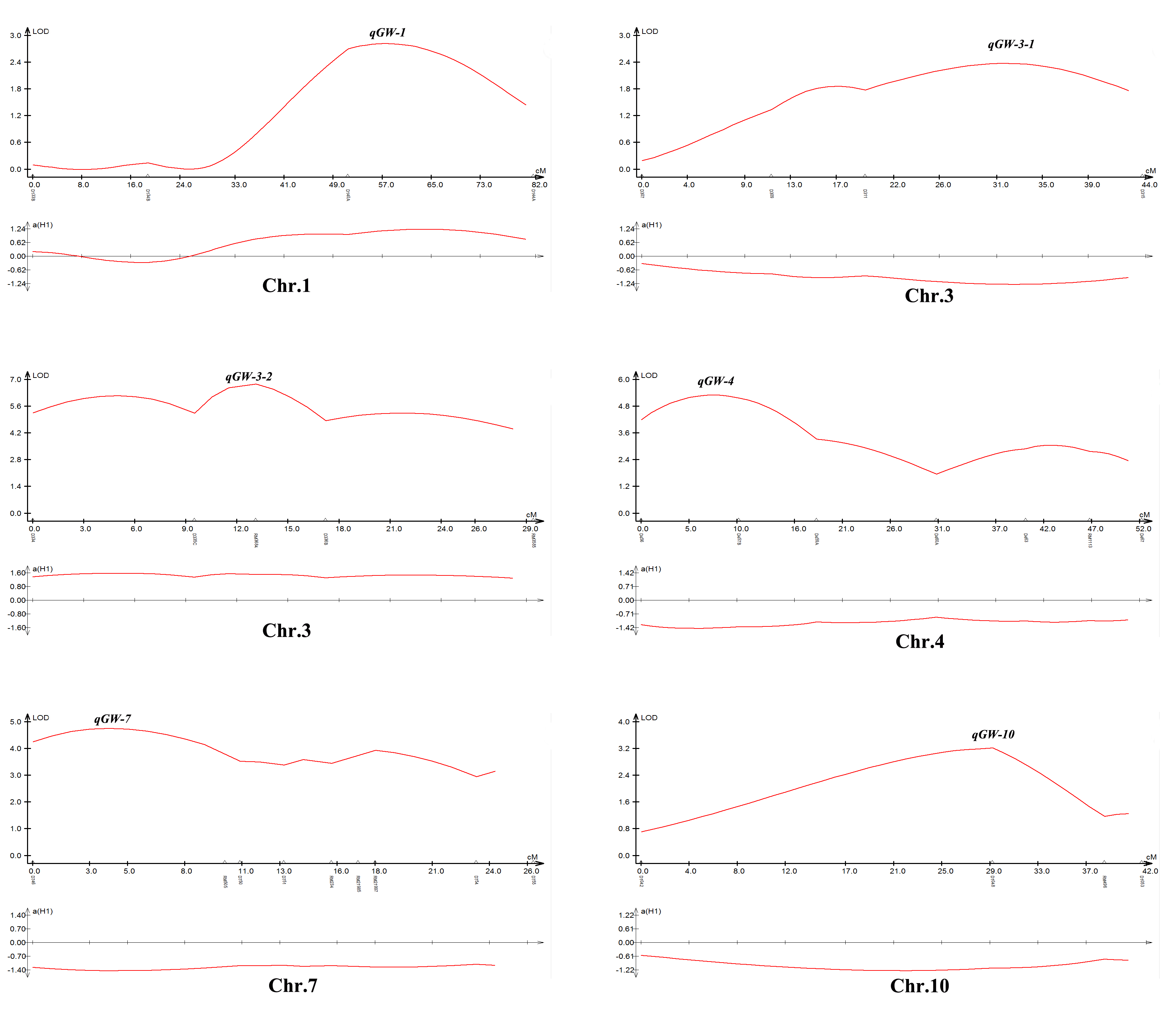

Supplement: S5 Fig — (TIF) [file pone.0181588.s005.tif]

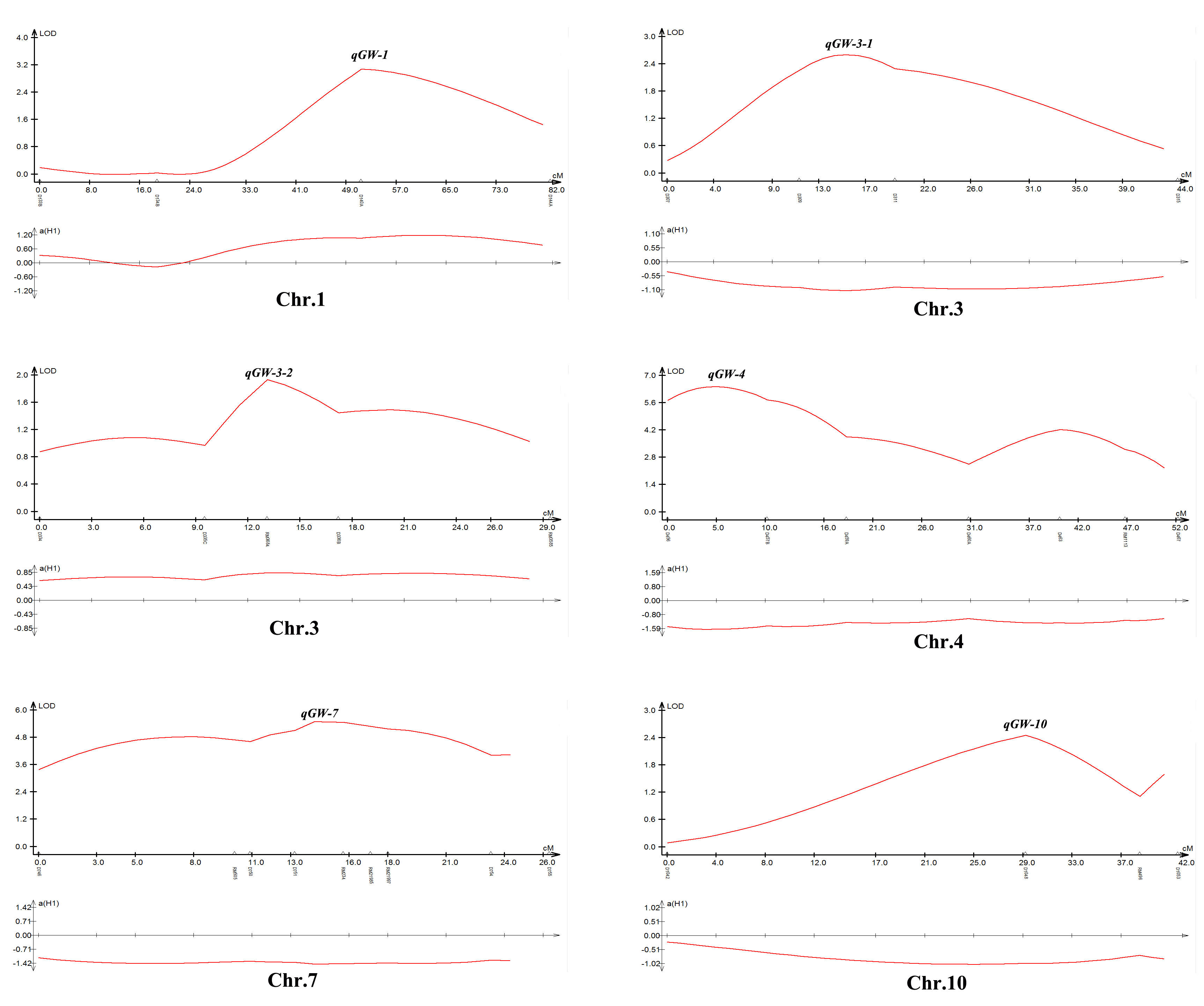

Supplement: S6 Fig — (TIF) [file pone.0181588.s006.tif]

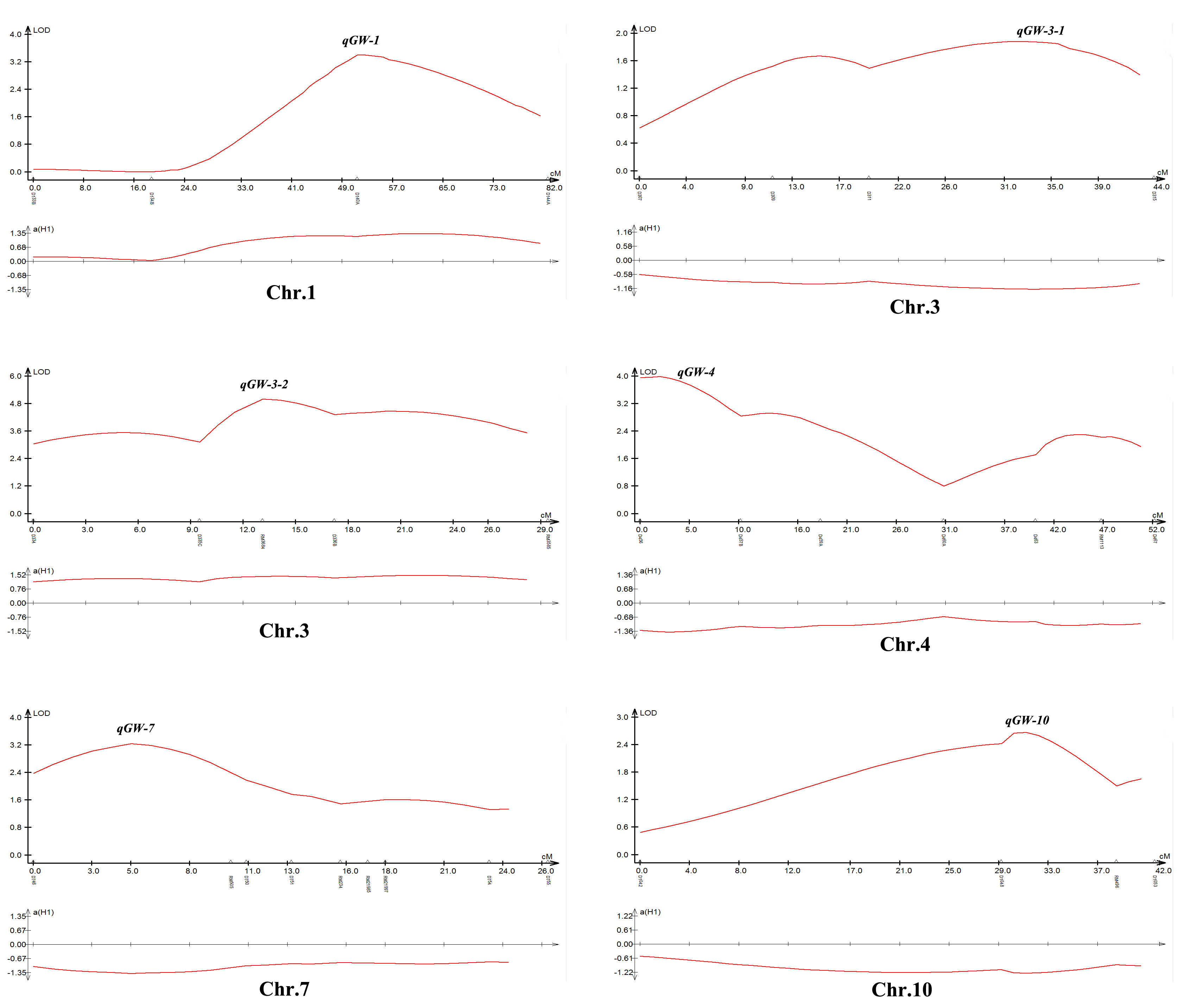

Supplement: S7 Fig — (TIF) [file pone.0181588.s007.tif]

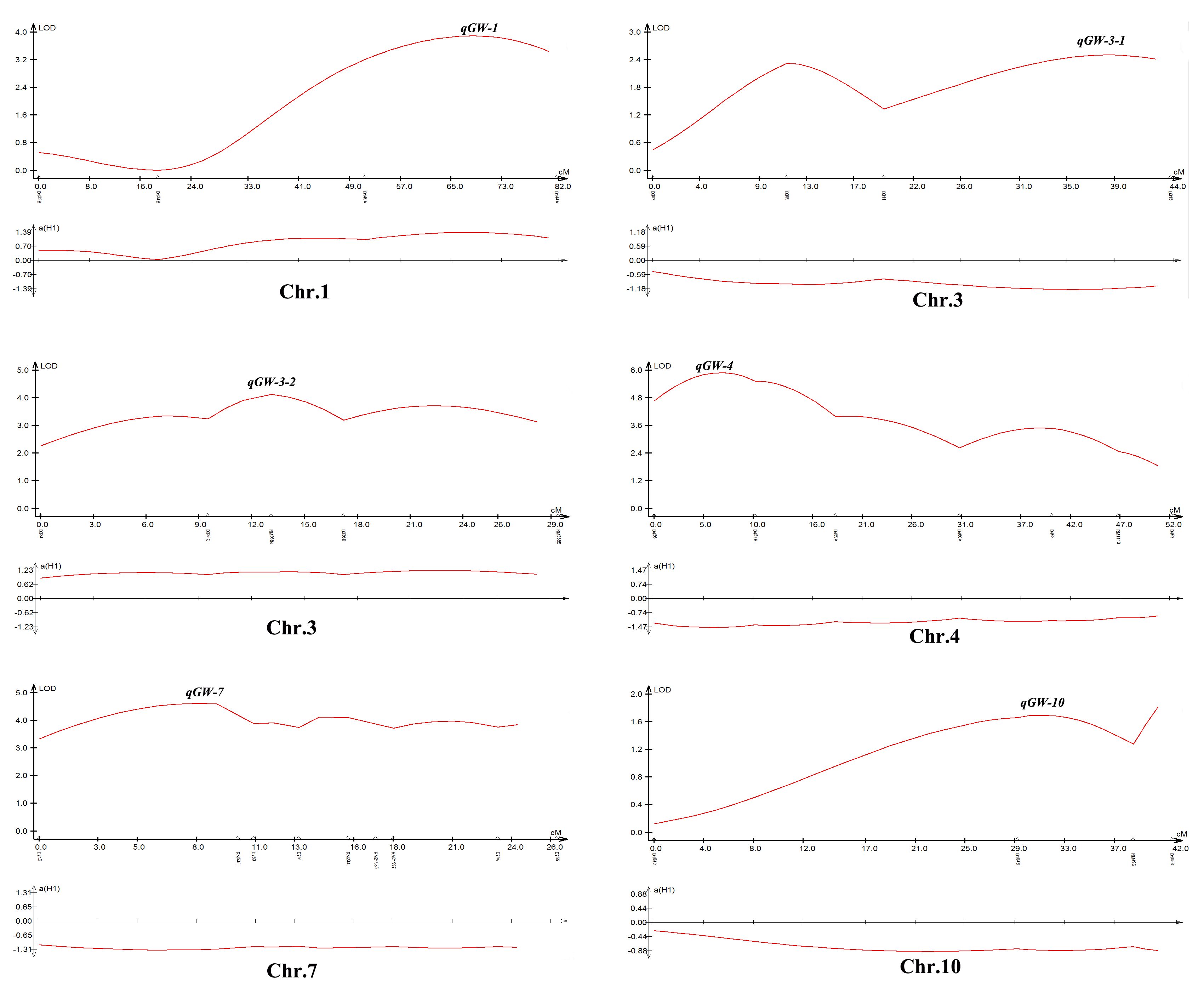

Supplement: S8 Fig — (TIF) [file pone.0181588.s008.tif]
