# Supplementary material for: Combination of twelve alleles at six quantitative trait loci determines grain weight in rice
Source: PLoS One. 2017 Jul 18;12(7):e0181588. doi: 10.1371/journal.pone.0181588 (PMC5515452; doi:10.1371/journal.pone.0181588)
Supplement: S1 Table — DF: degrees of freedom; SS: sum of squares; *, P < 0.05; **, P < 0.01. (DOCX) [file pone.0181588.s009.docx]

**S1 Table.** **Two-way analysis of variance used to confirm the digenic epistatic loci detected in the F_3_ population, derived from ‘Lemont’ × ‘Yangdao 4’, and grown in 2012 in Hainan, using inclusive composite interval mapping.**

|  | **DF** | **Type Ⅰ SS** | **Mean square** | **F value** | ***P*** |
| --- | --- | --- | --- | --- | --- |
| RM1201 | 2 | 5.62 | 2.81 | 0.56 | 0.57 |
| RM3404 | 2 | 4.28 | 2.14 | 0.43 | 0.65 |
| RM1201 ×RM3404 | 4 | 54.28 | 13.57 | 2.72 | 0.03* |
| D122E | 2 | 5.03 | 2.52 | 0.63 | 0.53 |
| D436 | 2 | 3.43 | 1.72 | 0.43 | 0.65 |
| D122E×D436 | 4 | 70.93 | 17.73 | 4.47 | 0.002** |
| D128A | 2 | 7.39 | 3.69 | 0.75 | 0.47 |
| D643 | 2 | 5.38 | 2.69 | 0.55 | 0.58 |
| D128A×D643 | 4 | 58.37 | 14.59 | 2.97 | 0.02* |
| D205 | 2 | 9.42 | 4.71 | 0.95 | 0.39 |
| D927 | 2 | 15.58 | 7.79 | 1.58 | 0.21 |
| D205×D927 | 4 | 54.11 | 13.53 | 2.74 | 0.03* |
| D301 | 2 | 9.19 | 4.60 | 0.94 | 0.39 |
| D933 | 2 | 5.92 | 2.96 | 0.61 | 0.55 |
| D301×D933 | 4 | 51.03 | 12.76 | 2.61 | 0.04* |
| D307 | 2 | 4.44 | 2.22 | 0.46 | 0.63 |
| D440 | 2 | 4.36 | 2.18 | 0.45 | 0.64 |
| D307×D440 | 4 | 64.78 | 16.20 | 3.34 | 0.01* |
| D1113 | 2 | 1.24 | 0.62 | 0.14 | 0.87 |
| D1133 | 2 | 3.23 | 1.62 | 0.37 | 0.69 |
| D1113×D1133 | 4 | 82.30 | 20.57 | 4.67 | 0.002** |

DF: degrees of freedom; SS: sum of squares; *, *P* < 0.05; **, *P* < 0.01.
