# Supplementary material for: Combination of twelve alleles at six quantitative trait loci determines grain weight in rice
Source: PLoS One. 2017 Jul 18;12(7):e0181588. doi: 10.1371/journal.pone.0181588 (PMC5515452; doi:10.1371/journal.pone.0181588)
Supplement: S2 Table — DF: degrees of freedom; SS: sum of squares; *, P < 0.05; **, P < 0.01. (DOCX) [file pone.0181588.s010.docx]

**S2 Table. Two-way analysis of variance used to confirm the digenic epistatic loci detected in the F_2_ population, derived from ‘Lemont’ × ‘Yangdao 4’, and grown in 2012 in Hangzhou, using inclusive composite interval mapping.**

|  | **DF** | **Type Ⅰ SS** | **Mean square** | **F value** | ***P*** |
| --- | --- | --- | --- | --- | --- |
| D1252 | 2 | 8.05 | 4.02 | 0.48 | 0.62 |
| D755 | 2 | 19.41 | 9.71 | 1.17 | 0.31 |
| D1252 ×D755 | 4 | 139.76 | 34.94 | 4.21 | 0.003** |

DF: degrees of freedom; SS: sum of squares; **, *P* < 0.01.
